# THE DEVELOPMENT AND IMPLEMENTATION OF A CARDIAC HOME HOSPICE PROGRAM FOR ADVANCED STAGE HEART FAILURE PATIENTS

**DOI:** 10.1093/geroni/igz038.2913

**Published:** 2019-11-08

**Authors:** Lizeyka Jordan, Ruth Masterson Creber, David Russell, Dawon Baik

**Affiliations:** 1 Visiting Nurse Service of New York, New York, New York, United States; 2 Weill Cornell Medicine, New York, New York, United States; 3 Appalachian State University, Boone, North Carolina, United States; 4 Columbia University, New York, New York, United States

## Abstract

Heart failure (HF) patients encounter distressing symptoms at the end-of-life including dyspnea, edema, and fatigue. Left untreated, these symptoms increase risk for hospice disenrollment. This presentation used the RE-AIM framework to examine a cardiac home hospice program for HF patients. Qualitative interviews with hospice providers (N=32) and quantitative medical record data were used to examine the program. Reach—1,183 HF participants were served between 2013-2016. Effectiveness—Enrollment of HF patients in the hospice program increased from 7.9% to 9.5% after the cardiac protocol was implemented. Adoption—implementation was spearheaded by a clinical champion aware of the challenges of HF symptom management. Implementation— additional support services (i.e., paramedicine, infusion services, cardiac comfort medication kits) were incorporated in the cardiac protocol to better manage complex clinical cases in the home. Maintenance— Reinforcing factors include ongoing training for nursing staff and a 3.5-hour introduction module providing information about HF case management and symptoms/treatments.

